# A novel approach toward cyberbullying with intelligent recommendations using deep learning based blockchain solution

**DOI:** 10.3389/fmed.2024.1379211

**Published:** 2024-04-02

**Authors:** Aliaa M. Alabdali, Arwa Mashat

**Affiliations:** ^1^Faculty of Computing and Information Technology, King Abdulaziz University, Department of Information Technology, Rabigh, Saudi Arabia; ^2^Faculty of Computing and Information Technology, King Abdulaziz University, Department of Information Systems, Rabigh, Saudi Arabia

**Keywords:** public health, prediction, health monitoring, blockchain, cyberbullying, federated learning, decision making

## Abstract

Integrating healthcare into traffic accident prevention through predictive modeling holds immense potential. Decentralized Defense presents a transformative vision for combating cyberbullying, prioritizing user privacy, fostering a safer online environment, and offering valuable insights for both healthcare and predictive modeling applications. As cyberbullying proliferates in social media, a pressing need exists for a robust and innovative solution that ensures user safety in the cyberspace. This paper aims toward introducing the approach of merging Blockchain and Federated Learning (FL), to create a decentralized AI solutions for cyberbullying. It has also used Alloy Language for formal modeling of social connections using specific declarations that are defined by the novel algorithm in the paper on two different datasets on Cyberbullying and are available online. The proposed novel method uses DBN to run established relation tests amongst the features in two phases, the first is LSTM to run tests to develop established features for the DBN layer and second is that these are run on various blocks of information of the blockchain. The performance of our proposed research is compared with the previous research and are evaluated using several metrics on creating the standard benchmarks for real world applications.

## 1 Introduction

Within the dynamic sphere of social media, the persistent issue of cyberbullying demands inventive and robust solutions to ensure user safety and cultivate a secure digital environment. Recent insights from the “Cyberbullying Statistics, Facts, and Trends (2023) with Charts” (1) underscore concerning statistics, revealing that over 61% of teens on social media have encountered online bullying related to their appearance, while 41% of adults have personally confronted harassment on social media. A thorough examination of cyberbullying rates among adolescents further underscores the gravity of the issue, with a study in England revealing an incidence of 17.9%, and research in Saudi Arabia reporting a prevalence of 20.97% (2). Despite recognized correlations between socio-economic factors, environmental influences, mental health, and cyberbullying tendencies, there remains an unexplored dimension—the creation of an online self-sufficient system to address cyberbullying and offer necessary guidance to identified victims and bullies.

As our digital interconnectedness expands, so too does the urgency to confront the challenges posed by malicious online behaviors. This paper proposes a novel approach to combat cyberbullying by integrating findings from cyberbullying statistics with innovative solutions. Our approach involves the fusion of two cutting-edge technologies: Blockchain and Federated Learning (FL) (3). Blockchain, known for its decentralized nature and transaction integrity, serves as the foundation of our solution, while Federated Learning facilitates collaborative machine learning without compromising individual data privacy. Alloy Language is utilized for the formal modeling of social connections, with specific declarations defined by our novel algorithm shaping the foundation of our proposed methodology. The incorporation of Long Short-Term Memory (LSTM) and Deep Belief Networks (DBN) into our system architecture enables established relational checks as well as feature detection within the DBN layer. Recognizing the importance of user accessibility, we augment our approach with an eXplainable Artificial Intelligence (XAI) layer, which sits atop our integration of Deep Learning and Blockchain technologies, making the solution more understandable to users in real-world circumstances. In the dynamic scenario of online interactions, natural language processing with AI capabilities emerges as an important aspect in the study of Cyberbullying, this plays an important role in developing useful features textual data. With the growth in usage of social media communication and utilization of day to day activities, prevalence of NLP with AI capabilities to study and analyze human interactions, innate sentiments, and discourse patterns has become increasingly relevant. The availability of vast amounts of data and the development of NLP and AI capabilities are the main drivers which cause the surge in the field of Sentiment Analysis, Tone detection etc. (4). The same is also used in fields such as information retrieval, topic modeling, sentiment analysis, and more. Cyberbullying has developed as a major issue in today’s socially connected generation, with reference to the purposeful and repetitive use of digital communication by miscreants to harass, intimidate, or hurt individuals. Cyberbullying includes a wide range of damaging activities such as spreading rumors, publishing sexual or slanderous content, sending abusive communications, and participating in online hate speech. Individuals’ mental health, social interactions, and overall well-being are all negatively impacted by cyberbullying (5).

The design is kept such that the proposed solution can be deployed using existing packaging and MLOps processes. The work explored in this document aims to contribute to the existing studies on detection and prevention of cyberbullying by proposing a novel approach and make online spaces safer. It combines three powerful technologies: federated learning, blockchain, and deep learning with natural language processing (NLP). Federated learning protects user privacy by training the cyberbullying detection model on individual devices without sharing the data itself. Blockchain ensures the security and tamper-proof nature of the training process. Deep learning and NLP enable the model to accurately identify cyberbullying content.

Through this Blackbox model powered by federated learning and NLP techniques, we develop a model that works primarily on two factors – Preservation of Social Media User Privacy and increasing the accuracy of Cyberbullying detection. The work done in this paper works in line with objective of creating safer online spaces by detecting cyberbullying and hence giving a boost to the mental health of individuals in the digital era. Our study follows a well-defined federated training sequence of various blocks, that has been developed to implement both user privacy and high-speed block chain based deep learning methods, toward cyberbullying detection.

In this paper, we have made the following contributions:

- To propose a novel framework using Blockchain and Federated Learning based Cybersecurity Solution (BFL-CS) to handle cyberbullying in social media space.
- To develop novel algorithms which works as a Hybrid Block Chain & Federated Learning model for the prevention Cyber bullying solution.
- To evaluate the proposed method with other deep learning-based methods, by using a dual layer deep learning architecture using LSTM and DBN techniques.
- To assess the effectiveness of the work using metrics and visualization tools.

The paper has been organized as follows: Section 1 discusses the Introduction and contributions made, Section 2 highlights the previous researches done in the field. Section 3 mentions the detailed proposed framework and methodology. Section 4 presents the evaluation and discussion of the results and last section concludes with some future directions.

## 2 Literature review

Muniyal el al. (3) introduced Federated Learning [FL] as a procedure to secure sensitive user data across the process pipeline. The authors emphasize more toward the possibility of a security breach on a Cyberbully detection and prevention system when the same is based on a Central Server. In addition to this, the performance parameters of the proposed solution is shown only on a IID (Independent and Identically Distributed) dataset only. The solution developed is named as “FedBully,” which used NLP techniques such as sentence-embedding based classifier, Sentence-BERT (Bidirectional Encoder Representations from Transformers) to detect cyberbullying, incorporating the training procedure from federated learning. Iwendi et al. (6) proposes a pure Deep Learning based solution for detection of Cyberbullying in Social Media. Advanced techniques like Bidirectional Long Short-Term Memory (BLSTM), Gated Recurrent Units (GRU), Long Short-Term Memory (LSTM), and Recurrent Neural Network (RNN) are used in ensemble to generate a higher accuracy – AOC (Area Under the Curve) for the proposed solution. In addition to that, the solution also does a significant amount of text cleaning and tokenization efforts. The paper also explores a comparative analysis of various other deep learning methods and provides a qualitative result of each method with respective accuracies and process performances. Samee et al. (2) showed detection of cyberbullying with federated learning. The work improved the identification of cyberbullying cases by offering a richer knowledge of the emotional context within communications by developing eight novel emotional elements retrieved from textual tweets. The use of privacy-preserving federated learning enabled collaborative cyberbullying detection, maintaining data privacy while encouraging collaboration across varied groups for a more scalable and successful method. Furthermore, similar to Iwendi et al. (2) where the analysis done in the paper used a client selection strategy for overall model ensemble preparation which was purely based on statistical performance of the model, the output was desired to be more accurate. The paper showed that the BERT model used in Gohal et al. (2) outperforms other traditional models such as CNN, DNN, and LSTM, that too with such low number of epochs, i.e., 200.

### 2.1 Research gap

Based on the literature review, we see that in previous research works on cyber-bullying detection and mitigation, a drawback that we constantly notice is the centralization of sensitive user data compared to social media for deep learning model training, highlighting a major privacy concern (12). This disadvantage may also make the adoption of such systems problematic when applied to real-world applications, as consumers will be hesitant to provide data with systems that take no precautions to safeguard their data (13). Furthermore, we show that traditional approaches frequently struggle to perform effectively due to a lack of different user behavior data and linguistic patterns. In our research, we effectively solve the above mentioned issues by combining federated learning with a secure block chain-based backend and alloy data modeling techniques. Federated learning uses a decentralized strategy to ensure that user data is handled and stored ensuring user privacy. Furthermore, the basic working of primary deep learning methods provides us with opportunity of continuous model tweaking, which, combined with other data security measures helps us in achieving our goal without giving away the third-party data security (7). Our paper uses features of federated learning to handle these shortcomings of earlier methodologies, resulting in a ground-breaking approach to cyberbully identification that maintains the highest level of user information privacy and data security.

### 2.2 Comparative study of systems proposed in earlier works

**Table:** 

#	Paper title & Ref No.	Advantages	Disadvantages	Techniques used	Dataset	Accuracy
1	Shetty et al. (3)	Masking of data at the before start of data preprocessing leading to higher data security	High run time, two fold increase in computational load on the system.	SBERT, Universal Sentence Encoders –DAN, Universal Sentence Encoders – Transformers	Data from Kaggle, Youtube, Twitter	97.12%
2	Fati et al. (8)	Data pre processing is made a part of Deep Learning Methodologies, leading to a more holistic output.	Running NLP and DL together leads to a dependency on one layer for processing the other layer. So, a failure in the NLP layer can make the entire architecture crumble.	Continuous Bag of Words based Conv1DLSTM	Data from Kaggle	97.34%
3	Bruwaene et al. (9)	Ensemble Deep learning method used to get take advantage of various model accuracies.	High run time and need to multiple steps of data preprocessing and encoding.	Multi-technique annotation and a ensemble of SVM, CNN & XGBoost	VISR Dataset	–
4	Bozyigit et al. (10)	Vanilla Artificial Neural Networks	Low accuracy of the model	Artificial Neural Networks	Twitter – Hindi/Marathi	91%
5	Samee et al. (11)	Federated Learning used with basic Machine Learning processes.	No emphasis is given on the security aspect of the deep learning layer.	FedBERT	Twitter	92.15%

## 3 Proposed design methodology

This paper envisages novel method named Blockchain based Federated Learning based Cybersecurity Solution (BFL-CS) methodology to handle cyberbullying in social media space and its prevention (14). In the approach defined in this study, a Federated Learning methodology is employed with methods such as a modified LSTM in tandem traditional DBN to improve on the statistical parameters of the model and the privacy security of the model. The LSTM has traditional parameters such as batch size, timesteps and input feature vectors. It is to be noted that the DBN model is used as per its usual implementation without any modifications.

The proposed methodology works on two layers of memory:

1. A short-term memory (LSTM) that helps in generating blocks and federated learning nodes.
2. A long term memory (DBN) that keeps the learning from federated learning nodes and propagates it across the model during future epochs.

In this way, the model achieves faster run time due to actively forgetting information that does not value the model in the long run. And also generates highly accurate results from its long memory model implementation.

In a classical Ensemble implementation, the accuracy of two or models is combined to get a unified result. However, in our model, we have two DL models working together on the same data but at different stages to generate a result.

**Figure 1:**
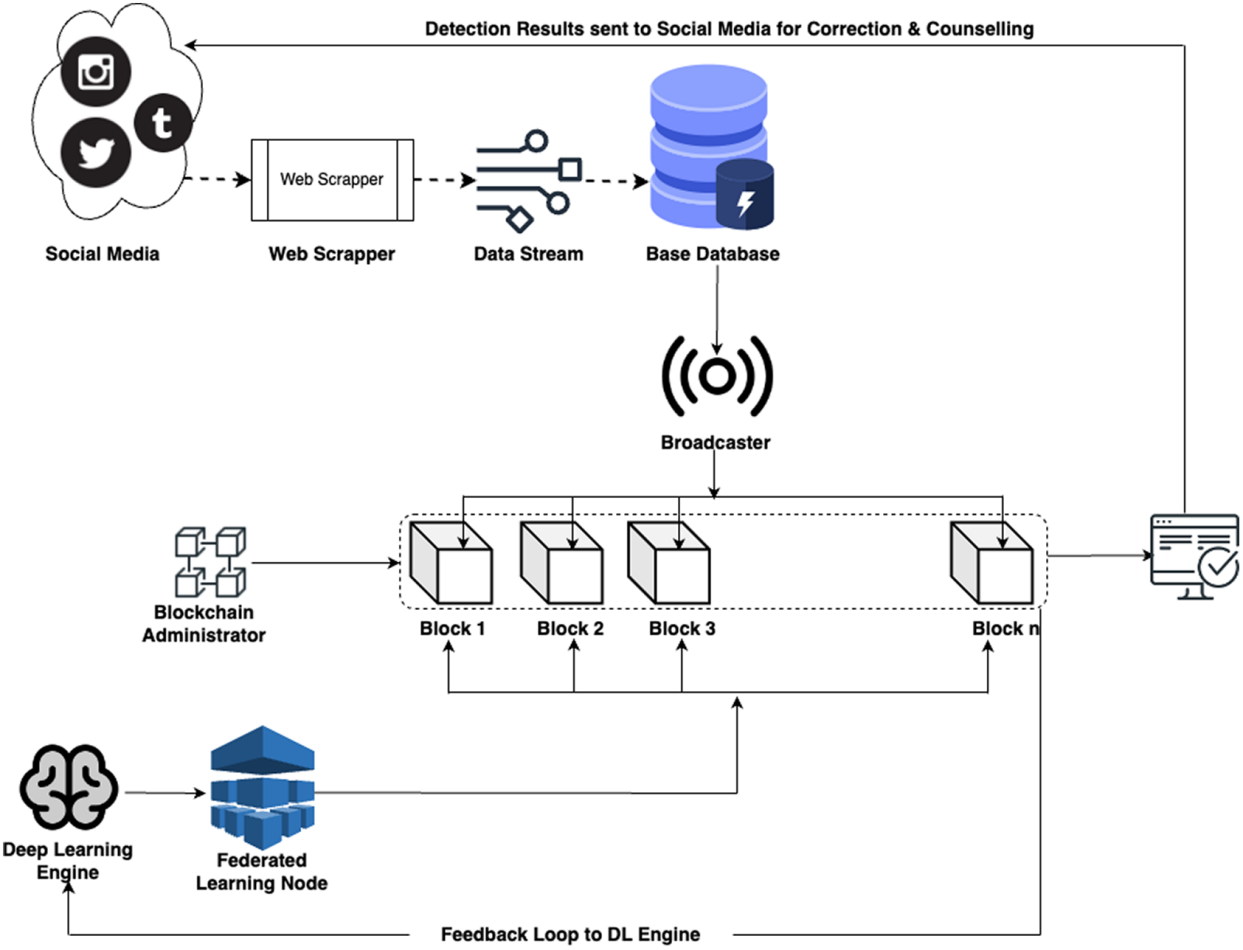
Overall structure of the BFL-CS model.

The architecture given below shows the complete data flow and working of the proposed design (Figure 1).

The framework model is listed and explained in the following steps.

### 3.1 Data warehousing

In our system architecture, the data is mainly collected from Social media platforms using Web Scrapping APIs. This scrapping is running on a preset scheduler to collect information at regular intervals of time and new data is added to the existing information set (15). In our model, data is stored in PostgreSQL. Currently the solution is hosted locally, however, as the complexity and size of data increases, we plan on scaling the solution toward AWS S3 with 3 AZs.

### 3.2 Data pre-processing

At this stage the data is made ready for ingesting into the model for obtaining desired performance. In the signal, it clears unnecessary effects, prevents issues, and improves accuracy. In this stage dataset namely the “BFL-CS dataset” and operations such as data cleaning, normalization and development of data stream is done.

### 3.3 Data cleaning and normalization

All the blank value fields and social media comments which clean word stems are not established are deleted from the database to prevent any kind of influence on the model due to high level outliers. Also, in order to eliminate the influences presented in the dissimilar scale features is executed in this process which reduces the model’s run time.

In many cases in the data science space, data scientist use the method of min-max normalization process. However, this method has its own problems, since this is rather a feature scaling method – this normalization significantly lowers the biasness of the model. While a lot of cases see biasness as a vice, in our case the biasness of the model actually points us toward the habitual bullies (16). Therefore, in our model we apply a rather lesser known normalization process which creates a correlation between the dataset and the standard deviation of the dataset.

We use the Z -Score normalization procedure to normalize the data and scaling it as per the requirement of the proposed model shown in Equation 1,

(1) $$x^{\#}=\frac{x-\bar{x}}{\sigma }$$

Here, $x^{\#}$ is the Z-normalized value. $\bar{x}$ is the average value/mean and σ is the standard deviation of the data. This normalization is used for all numeric data straight away and for the non-numeric data, the data is first undergone a one-hot encoding or normal encoding for normalization process.

### 3.4 Data stream for real time data publication to base database

This step involves a sophisticated integration of advanced data streaming and storage methodologies, as this step is very crucial in sensing repeated offenders and sensing their patterns. The various concepts incorporated in the model are as follows:

Event-Driven Architecture is a process that enables real-time processing by triggering and responding to events as they occur via web hooks, making it instrumental in capturing and handling data streams in real time. Kafka facilitates the building of real-time data pipelines and streaming applications. The process of collecting and importing real-time data streams into the base database for immediate storage and analysis. Utilizing messaging protocols (such as AMQP and XMPP) that minimize the time it takes for data to travel from source to destination, ensuring low-latency data delivery.

### 3.5 API Integration

Representational State Transfer APIs follow a set of architectural principles for designing networked applications, providing a standardized way for systems to communicate (17). Webhooks enable real-time communication between systems by triggering events in one system based on actions or updates in another, enhancing the responsiveness of API integrations. OAuth is a protocol for secure API authorization, allowing applications to access resources on behalf of a user with limited permissions. A centralized entry point that manages and optimizes API requests, ensuring scalability, security, and efficient data flow between systems. The entire design is parametric in nature without any hardcoded values. These parameters will be controlled by API driven microservices.

### 3.6 Data broadcaster to blockchain

At this stage a data broadcaster is developed which pushed the information to the blockchain, marrying the real-time dissemination of information with the immutable, decentralized characteristics of blockchain technology.

Key Components and Technical Processes involved at this stage. The deployment of a specialized protocol, such as DBP (hybrid ICMP & POP3), facilitates the secure and efficient real-time broadcasting of diverse data types onto a blockchain network. Decentralized Ledger Technology ensures a decentralized and distributed ledger, eliminating single points of failure and fortifying data availability across a network of nodes. The integration of a sophisticated execution engine ensures the seamless automation and enforcement of predefined rules embedded within smart contracts associated with the broadcasted data. The utilization of cryptographic hash functions, which is SHA-512 (specialized for our application), safeguards the immutability of data on the blockchain, rendering each block impervious to unauthorized modifications. The consensus algorithm, like Proof of Work (PoW) or Proof of Stake (PoS), orchestrates the agreement among network nodes, validating transactions and solidifying the security of the data broadcasting process. Blockchain’s inherent transparency provides an audit trail that allows participants to scrutinize the origin, journey, and modifications (if any) made to the broadcasted data, fostering accountability and trust. The comprehensive security architecture ensures the resilience of the data during transmission and storage, encompassing encryption, public-key infrastructure (PKI), and other robust security measures.

### 3.7 Blockchain administration system

This system tracks that individual changes are meticulously recorded within blocks, contributing to a transparent and tamper-resistant ledger with time & pseudo random number based identification module. The system allows for individual data entries to be added to the blockchain, with each piece of information forming a block in the distributed ledger. This decentralization eliminates the need for a central authority, enhancing transparency and reducing the risk of single points of failure (18). The heart of blockchain’s power lies in its unchangeability. Information in a block, once added, is cryptographically secured, making it virtually impossible to modify or erase. This feature guarantees the integrity of the recorded data throughout its entire existence. Every block in the blockchain is timestamped, providing an accurate record of when each data addition occurred. This temporal dimension adds another layer of transparency and traceability to the administration system. Smart contracts, self-executing contracts with predefined rules, can be incorporated to automate specific administrative functions. This enhances efficiency and reduces the need for manual intervention in routine processes. The administration of the blockchain is distributed across network nodes, eliminating the need for a centralized administrator. This decentralized governance model aligns with the principles of autonomy and inclusivity.

### 3.8 Deep learning engine

The deep learning engine that we are using in our architecture has two methods built in it. We first run classifications using LSTM and then we run another classification using Deep Belief Networks which then throws out the result.

Long Short-Term Memory (LSTM) is modified process of recurrent neural network (RNN) architecture designed to address the diminishing gradient situations in usual RNNs, enabling more effective modeling of sequential data. The key innovation of LSTMs lies in their memory cells, which allow them to capture and store information over long sequences.

Mathematically, as per theory, the following is to be noted in terms of LSTM model:

The base model contains of three units—the input unit ip , forget unit f , and output unit op .

In addition to that, data state is stored in – cell state cs .

The input unit handles the process flow of new information into the cell,

The forget unit controls the retention of existing information,

and the output unit handles the knowledge to be output from the cell.

The computations within an LSTM cell are governed by the following Equations 2–11:

(2) $$ip=RELU\left(Wt_{ii}ix_{t}+b_{ii}+Wt_{hi}h_{t-1}+b_{hi}\right)$$

(3) $$f=RELU\left(Wt_{if}ix_{t}+b_{if}+W_{hf}h_{t-1}+b_{hf}\right)$$

(4) $$op=RELU\left(Wt_{io}ix_{t}+b_{io}+W_{ho}h_{t-1}+b_{ho}\right)$$

(5) $$g=RELU\left(Wt_{ig}ix_{t}+b_{ig}+W_{hg}h_{t-1}+b_{hg}\right)$$

(6) $$cs=f_{t}\;X\;C_{t-1}+i_{t}\;X\;g_{t}$$

(7) $$h=o_{t}⊙\tan hC_{t}$$

Here, $ix_{t}$ is the input at time t , h is the hidden state at time t , RELU denotes the sigmoid activation function, and X represents element-wise multiplication. The weight multidimensional matrix Wt and bias column matrix b are parameters learned during the training process. The LSTM’s ability to selectively retain and utilize information over varying time intervals makes it well-suited for tasks involving sequential and time-series data.

In addition to LSTMs, the model proposed in the paper also used Deep Belief Networks (DBNs) in tandem.

Deep Belief Networks (DBNs) are a type of generative neural network architecture composed of multiple layers of stochastic, latent variables. DBNs consist of two main components: a stack of Restricted Boltzmann Machines (RBMs) and a top layer that serves as a discriminative model. The hidden layer of each RBM serves as the visible layer for the next, creating a hierarchical structure. The mathematical formulation of DBNs involves the activation probabilities of the hidden and visible layers, weight matrices, and biases. Let h represent the hidden layer and v the visible layer. The activation probabilities $P\left(h_{j}=1\right)$ for hidden unit j and $P\left(v_{j}=1\right)$ for visible i unit are given by:

(8) $$P\left(h_{j}=1\right)=\sigma \left(b_{j}+\sum_{i=1}^{N}W_{ij}v_{i}\right)$$

(9) $$P\left(v_{i}=1\right)=\sigma \left(c_{i}+\sum_{j=1}^{N}W_{ij}h_{j}\right)$$

Now we see that the algorithm that we are using inside our Deep Learning engine is a mixture of two base models. Therefore, the integration of Long Short-Term Memory (LSTM) and Deep Belief Networks (DBN) in a unified system leverages the strengths of both models enhances the modeling accuracies and generation of sequential data. In this hybrid system, the LSTM component helps in capturing long-term dependencies and patterns in sequential information, while the DBN component contributes to hierarchical feature learning and generation (19). Mathematically, the output of the LSTM ( $O_{LSTM}$ ) and DBN ( $O_{DBN}$ ) components can be combined to produce the final system output ( $O_{FINAL}$ ) as follows (20):

(10) $$O_{FINAL}=\alpha .O_{LSTM}+\left(1-\alpha \right).O_{DBN}$$

Here, α is a weighting parameter that determines the influence of each component on the final output. This hybrid approach aims to exploit the complementary strengths of LSTM and DBN, providing a more robust and expressive model for tasks such as sequence generation, where capturing both short-term and long-term dependencies is crucial. The choice of α allows for flexible adjustment of the contribution of each component, enabling fine-tuning based on specific task requirements and data characteristics.

### 3.9 Mathematical model

Consider the continuous-time outputs $O_{LSTM}\left(t\right)$ and $O_{DBN}\left(t\right)$ from the LSTM and DBN components, respectively. The continuous-time final output $O_{FINAL}\left(t\right)$ is expressed as an integral over time, with a parameterized blending factor α(t) denoting the time-varying contribution of each component shown in Equations 11–14:

(11) $$O_{FINAL}\left(t\right)=\int_{o}^{t}\left[\alpha \left(\tau \right).O_{LSTM}\left(\tau \right)+\left(1-\alpha \left(\tau \right)\right).O_{DBN}\left(\tau \right)\right]d\tau \;$$

This integral formulation captures the continuous evolution of the system’s output over time, reflecting the dynamic nature of the blending process.

The objective function for continuous-time training is defined as the integral of the squared error between the system’s output $O_{FINAL}\left(t\right)$ and the target output Y(t) :

(12) $$J\left(\emptyset_{LSTM},\emptyset_{DBN}\right)=\frac{1}{2}\overset{T}{\underset{0}{\int }}\left(O_{FINAL}\left(t\right)-Y{\left(T\right)}^{2}\right)dt$$

The gradients with respect to the parameters ( $\frac{\partial J}{\partial \emptyset_{LSTM}}$ and $\frac{\partial J}{\partial \emptyset_{DBN}}$ ) guide the continuous-time parameter updates during the training process.

The continuous-time optimization involves adjusting the parameters through an integral-based gradient descent approach:

(13) $$\emptyset_{LSTM}^{t+\Delta t}=\emptyset_{LSTM}^{t}-\eta \int_{t}^{t+\Delta t}\frac{\partial J}{\partial \emptyset_{LSTM}}d\tau$$

(14) $$\emptyset_{DBN}^{t+\Delta t}=\emptyset_{DBN}^{t}-\eta \int_{t}^{t+\Delta t}\frac{\partial J}{\partial \emptyset_{DBN}}d\tau \;$$

Here, η represents the learning rate, and Δt signifies the time step in the continuous-time parameter space during each iteration.

From the above mathematical model, we define a base algorithm on directions of which the entire architecture is built, the algorithm is as follows:

#### ALGORITHM 1 : Deep learning engine of BFL-CS.

**Table:** 

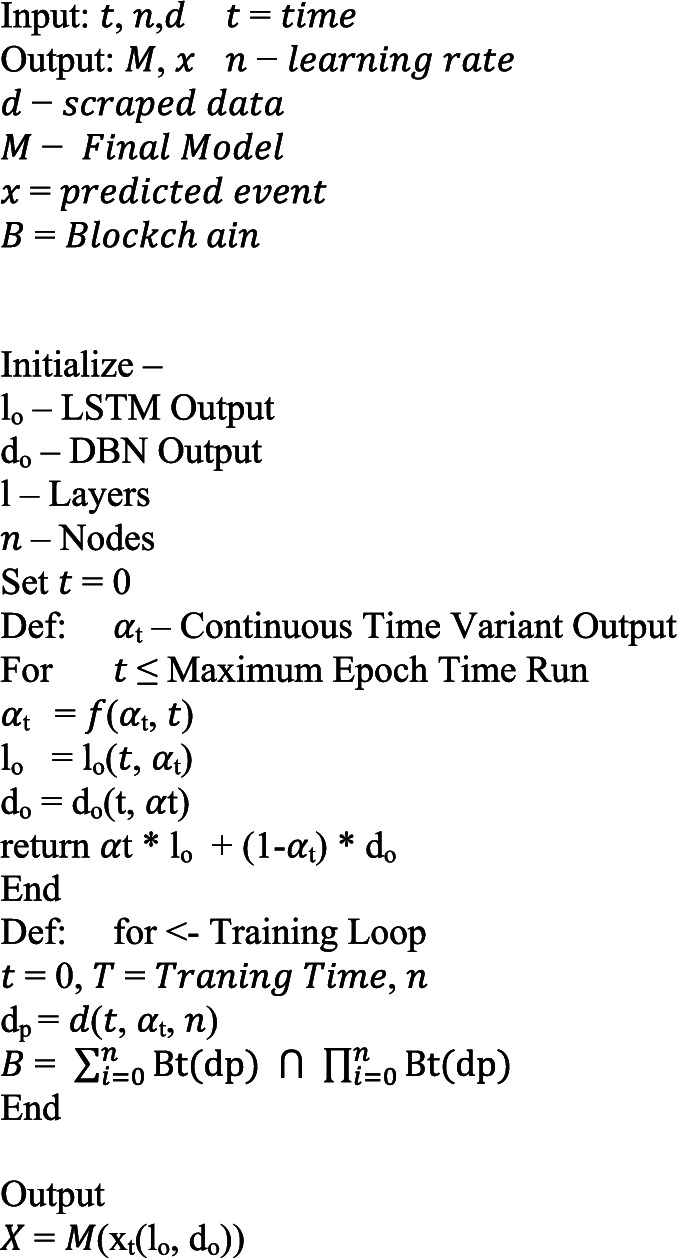

### 3.10 Federated learning node

In the context of the proposed model on combating cyberbullying through a decentralized defense system, Federated Learning (FL) emerges as a main technology backbone of the solution. By distributing the model training process across individual devices, FL ensures that sensitive user data, integral to understanding and mitigating cyberbullying, remains localized. The use of Federated learning is used to handle separate learning activities across the data. This step has actually made the system faster by running complex algorithms across small scale datasets with limited features.

This decentralized approach mitigates privacy concerns associated with centralization, a critical consideration in the realm of cyberbullying detection. Moreover, FL’s iterative model refinement, conducted collaboratively while preserving individual data, holds significant promise in enhancing the system’s understanding of evolving cyberbullying patterns. The incorporation of FL in the proposed system aligns with the broader goal of empowering users and institutions to actively contribute to the development of robust cyberbullying detection models, fostering a collective defense against online harassment while respecting individual privacy. The Algorithms 1, 2 for the complete model is given below:

#### ALGORITHM 2 : Complete BFL-CS Model.

**Table:** 

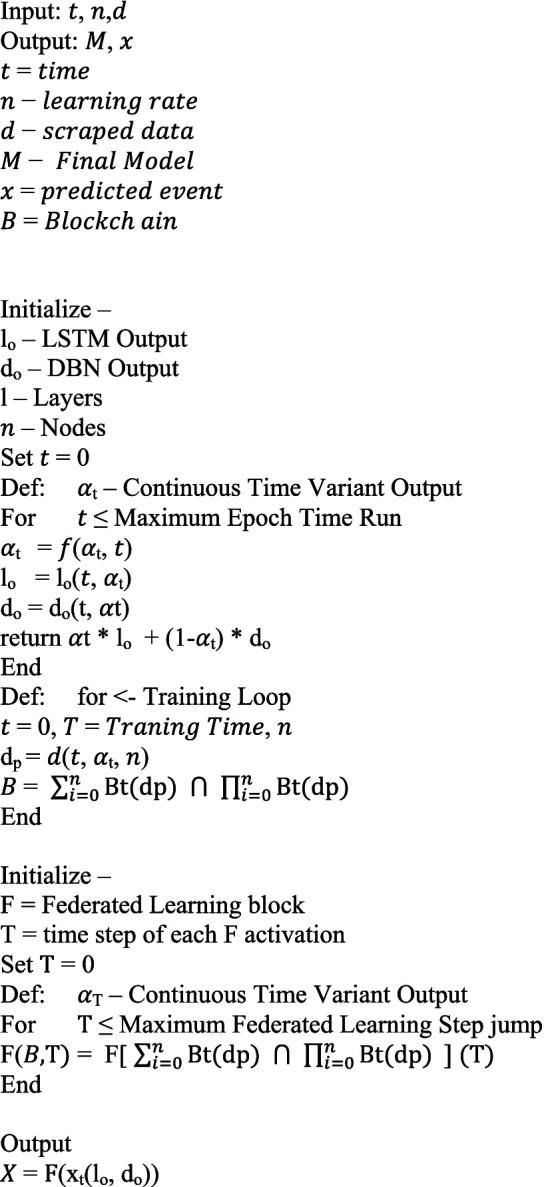

This code implements a secure federated learning system for training a combined LSTM and DBN model. In each round, clients are chosen to participate. They receive a global model, train their local versions on their own data, and calculate updates. To protect privacy, these updates can be masked with noise or securely combined before being sent back to a central server. The server aggregates the updates and improves the global model. Finally, for tamper-proof tracking, each improved model is recorded on a blockchain ledger. This process repeats for multiple rounds, resulting in a collaboratively trained model without ever sharing the raw data from individual clients.

### 3.11 Result node, feedback loop: to deep learning engine and corrective data loop: to social media

In the proposed system, the culmination of federated learning, LSTM, DBN, data collection, preprocessing, and blockchain management converges at the result node (21).

This node serves as the repository for the outcomes of the intricate processes conducted during each communication round. Subsequently, these results are broadcasted into the system feedback loop, initiating a sequence of actions for system parameter optimization. The system feedback loop strategically utilizes the obtained results to refine global model parameters, enhancing the overall effectiveness of the cyberbullying detection system. Simultaneously, the results are channeled into the social media loop, triggering actions against systemic bullies. This dynamic loop interfaces with social media platforms to deploy measures aimed at curtailing cyberbullying activities. The feedback-driven optimization process and decisive actions against online aggressors collectively contribute to the robustness and adaptability of the decentralized defense system, fostering a safer and more secure online environment.

### 3.12 Alloy modeling

In this paper, Alloy language helps in formalizing and modeling the intricate social connections within the context of cyberbullying detection (22). Alloy, a declarative modeling language, provides a robust framework for expressing and analyzing complex relationships between entities in a system. Specifically, we employ Alloy language to create formalized declarations and constraints that define the features and dynamics of social interactions within the cyber realm (23, 24). We construct a formal model that captures the essential features and constraints relevant to cyberbullying scenarios. This model helps in shaping the foundation of our proposed methodology, influencing the design of our novel algorithm. Alloy’s ability to articulate intricate relationships and constraints enhances the precision of our modeling efforts, contributing to the overall effectiveness of the decentralized defense system against cyberbullying.

## 4 Experimental results and discussions

The working of the BFL-CS method for detection and prevention of Cyberbullying in social media is tested with the Federated Deep Learning Processes which employ various methods.

The method is tested against various measures such as Recall, F1, Accuracy etc., and the results are compared with existing methods such as Vanilla RNN (v-RNN), Deep Reinforcement Learning (DRL), Residual Networks (ResNet) and Capsule Networks (CapNets). It is to be noted that the design is specifically made for English language analysis, it is seen that with appropriate data training, the results on various regional languages also show same results as shown by Pawar et al. (25) and Haider et al. (26).

### 4.1 Experimental setup

In this paper, the proposed methodology is implemented using Python and R. Pre-built packages are used for the implementation (27).

Details of the experimental setup along with the details of packages used are as follows:

**Table:** 

#	Particular	Specification/details
1	Processor	I7-14700K
2	RAM	8 GB
3	Operating Clock Frequency	3.6 GHz
4	IDE (Python)	PyCharm
5	IDE (R)	R Studio
6	Packages (Python)	TensorFlow, Caffe
7	Packages (R)	TensorFlow, H_2_O

### 4.2 Programming setup parameters

The performance of the proposed methodology is tested/implemented using hardware and software of the following specifications.

**Table:** 

#	Parameters	Specification/details
1	Training Epochs per Run	1,00,000
2	Dataset batch size	300
3	Learning Rate	0.001
4	Activation Function	Sigmoid
5	No. of Hidden Units	50
6	No. of Neurons Per Layer	10
7	Drop Out Rate	0.1
8	Loss Function	MSE

In this research, Mean Squared Error (MSE) serves as the error function, while the RELU activation function is employed.

The rate of learning is set to 0.001, with a bundle size of 300 and a dropout rate of 0.1. To enhance the performance of the BFL-CS method, a Gradient-based target optimizer is applied, as illustrated in Eqs. 12– 14 , for hyperparameter optimization in this study (28). Another important aspect is that the data is purely textual in nature (29).

### 4.3 Dataset description

In the paper, we have utilized the dataset of Cyberbullying which is available on Kaggle by Sahane et al. (30) & KLEJ (*Kompleksowa Lista Ewaluacji Językowych*) (31) to implement the BFL-CS method for detection of Cyberbullying.

There 48,000 data points are that we have collected from both (30, 31). The description is given below:

**Table:** 

#	Description	Detail
Data Source	(28, 29)	Available online
No. of Data Points	48,000	–
No. of Columns	4	Source of Data [twitter, youtube, tumblr], Tweet [40 Char], Date Time [DDMMYYYY HHMMSS], Location [Country]

### 4.4 Evaluation measures

The performance of the proposed method for Cyber bullying is evaluated through evaluation statistics such as Recall, Accuracy, Specificity, F1-score, etc. (32). The performance evaluation of these metrics is based on the mathematical expressions mentioned below.

*Accuracy:* This is the measure that measures the efficacy of the model with respect to correct classification of data-points on Cyberbullying scope.

*Precision:* This is the measure that shows the overall consistency of the model and shows how many instances does the model provide accurate classifications (12).

*Recall:* This measure shows the number of positive values that are measured on a random basis from the total number of positive classifications feedback (13).

*F1-score:* This is a derived value which is the mixture of Recall and Precision – basically the Harmonic mean of both these functions (33).

*Specificity:* This is again a very simple measure which sort of is the opposite of precision. This is the total negative hits of the model out of the total negative values (34).

### 4.5 Performance analysis

The statistical performance evaluation of the proposed model for detection and prevention of Cyberbullying in social media is tested with the Federated Deep Learning Processes which employ various methods.

**Figure 2:**
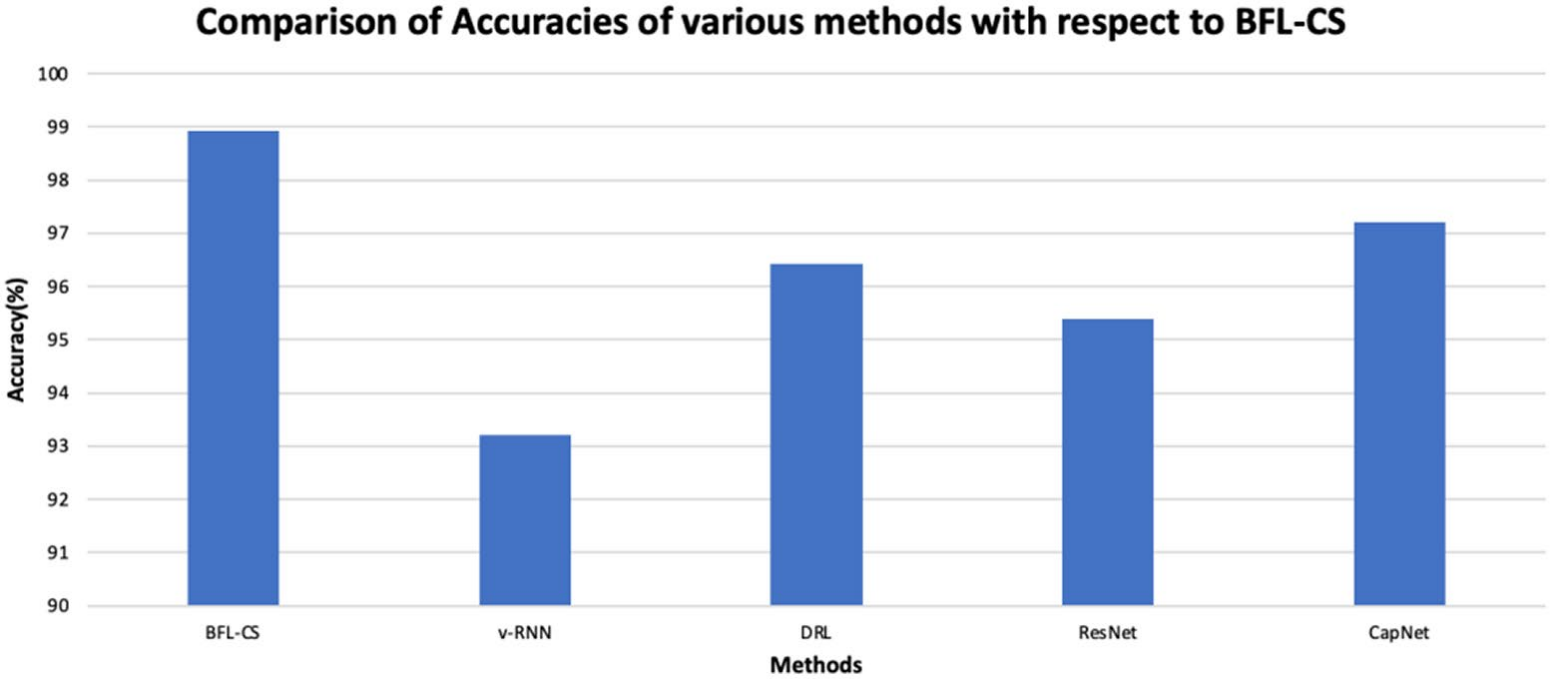
Comparison of accuracy.

**Figure 3:**
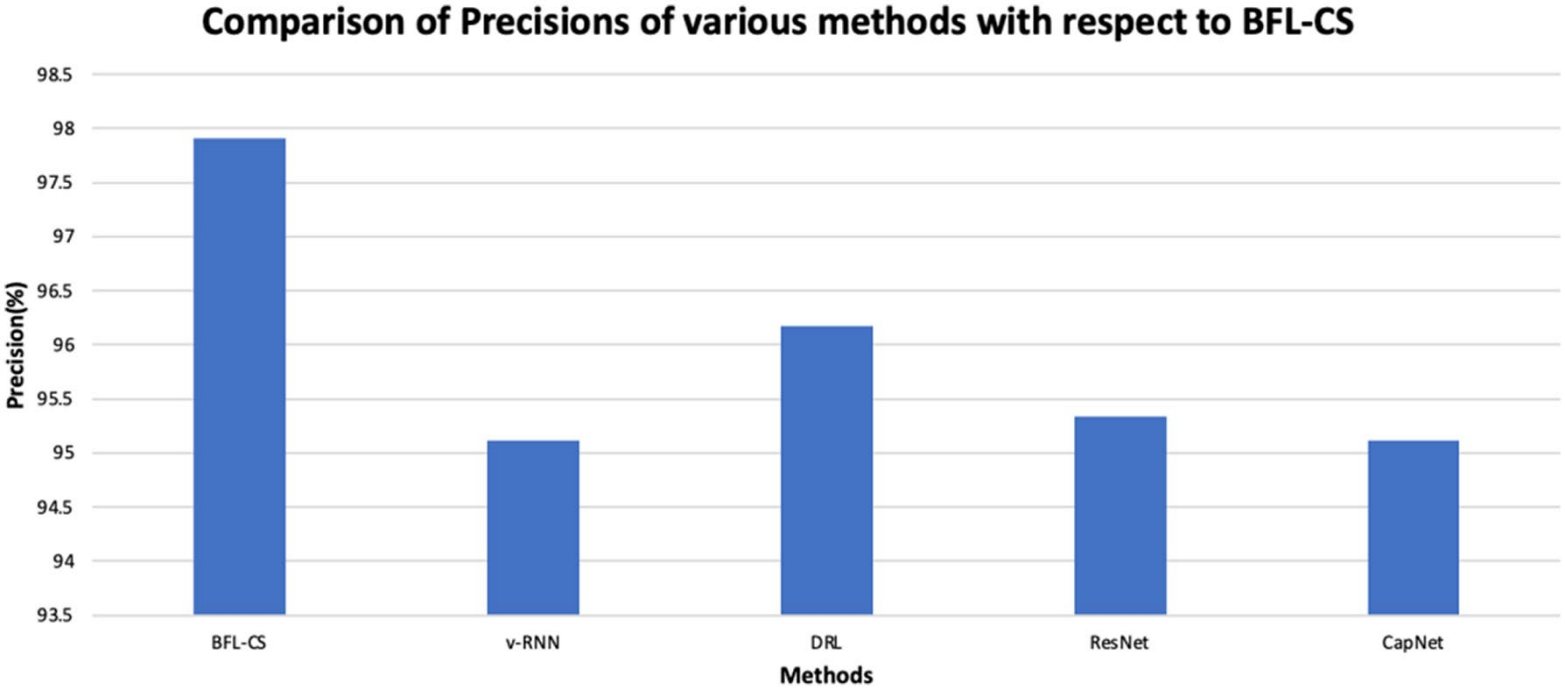
Comparison of precision.

**Figure 4:**
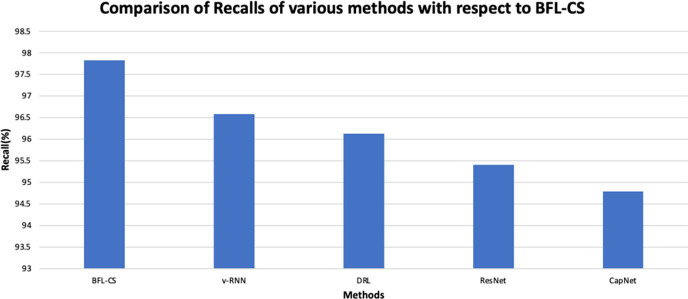
Comparison of recall measure.

**Figure 5:**
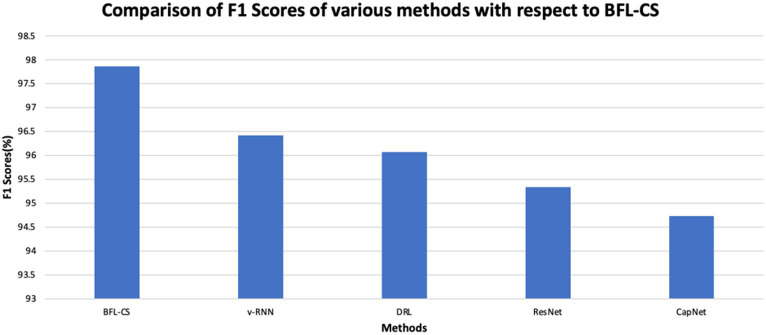
Comparison of F1-score.

**Figure 6:**
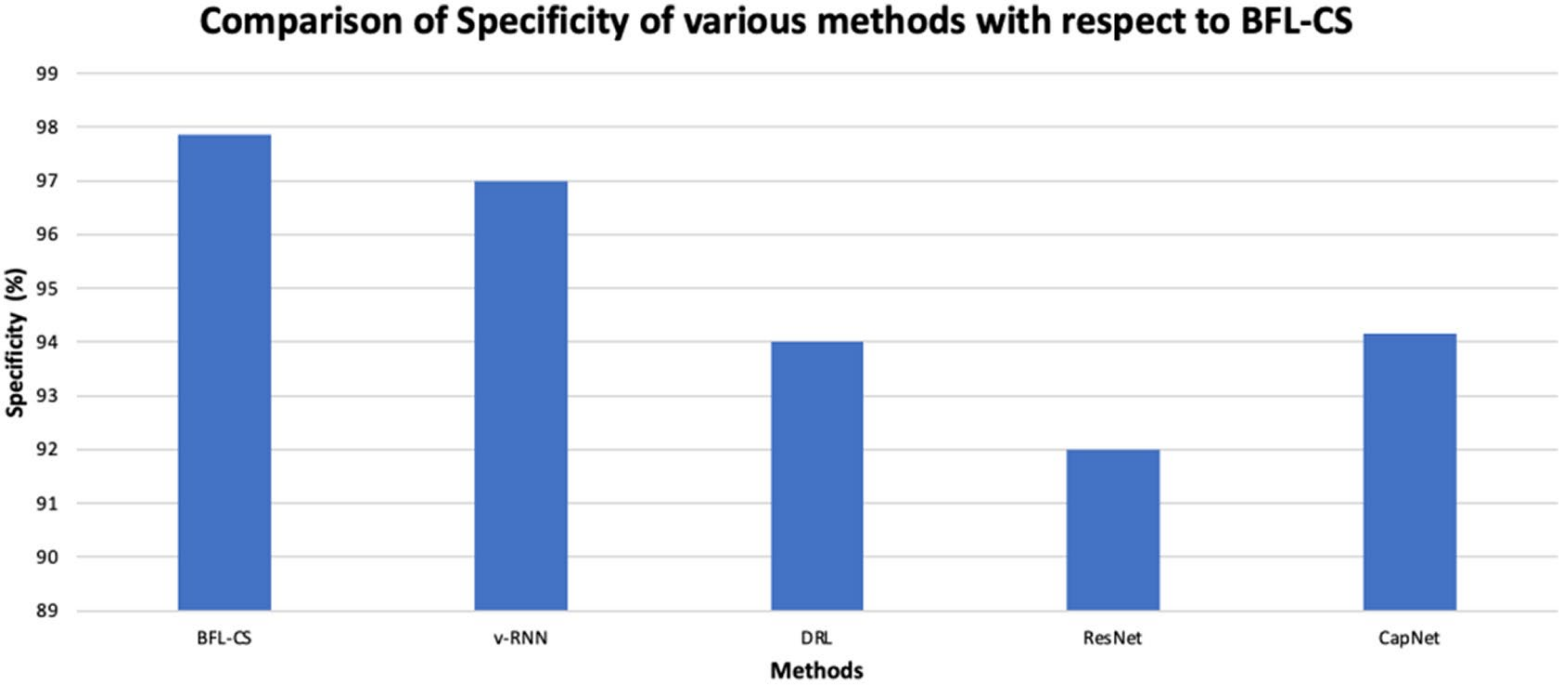
Graphical representation of specificity analysis.

The BFL-CS method is evaluated with various evaluation measures against existing methods such as Vanilla RNN (v-RNN), Deep Reinforcement Learning (DRL), Residual Networks (ResNet) and Capsule Networks (CapNets) (27, 35). From Figures 2–6, the performance of various methods as mentioned above are compared with respect to the BFL-CS. It is pertinent to note that the results are with respect to the overall accuracy of detection (36).

The plot shown in Figures 2, 3 illustrates the accuracy and precision of the BFLCS method in comparison to other known models. Impressively, BFLCS method has managed a remarkable accuracy of 98.92%. In contrast, established methods like v-RNN, DRL, ResNet, and CapNet demonstrated lower accuracies, recording values of 93.21, 96.43, 95.38, and 97.20%, respectively. Furthermore, examining precision, the BFL-CS method excels with a notable precision score of 97.91%.

We see that the system shows that it has achieved a high recall of 97.61% while the existing methods show much less recalls.

Figure 5 represents the graphical analysis to illustrate the F1-score of the BFL-CS method and the existing methods and again the superiority of the proposed solution.

**Figure 7:**
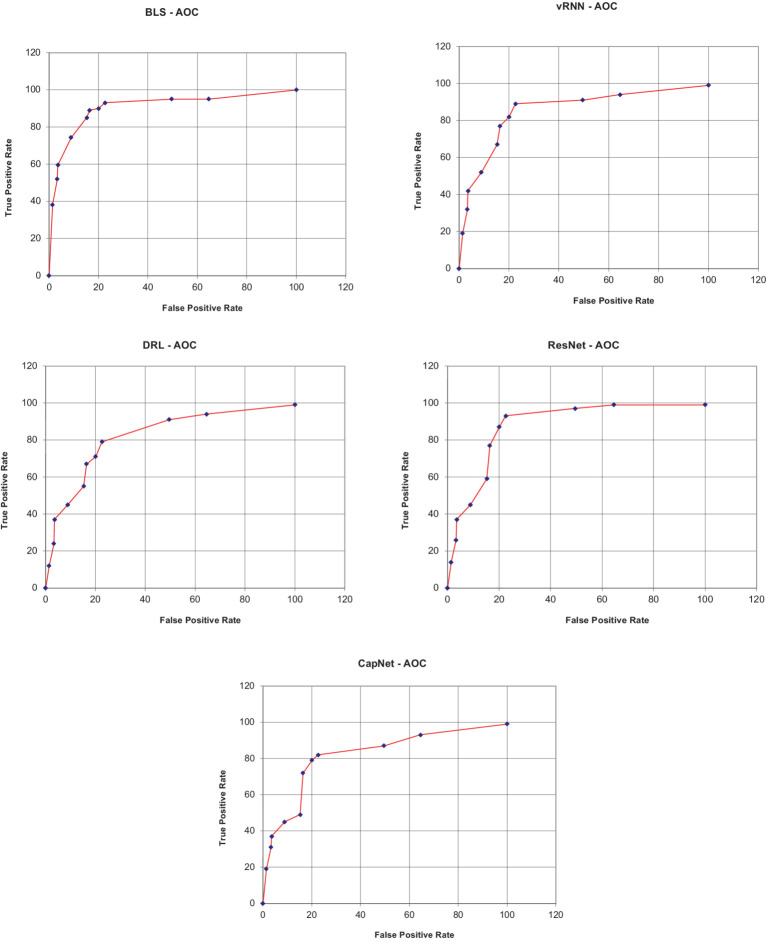
AUC-ROC plots.

**Figure 8:**
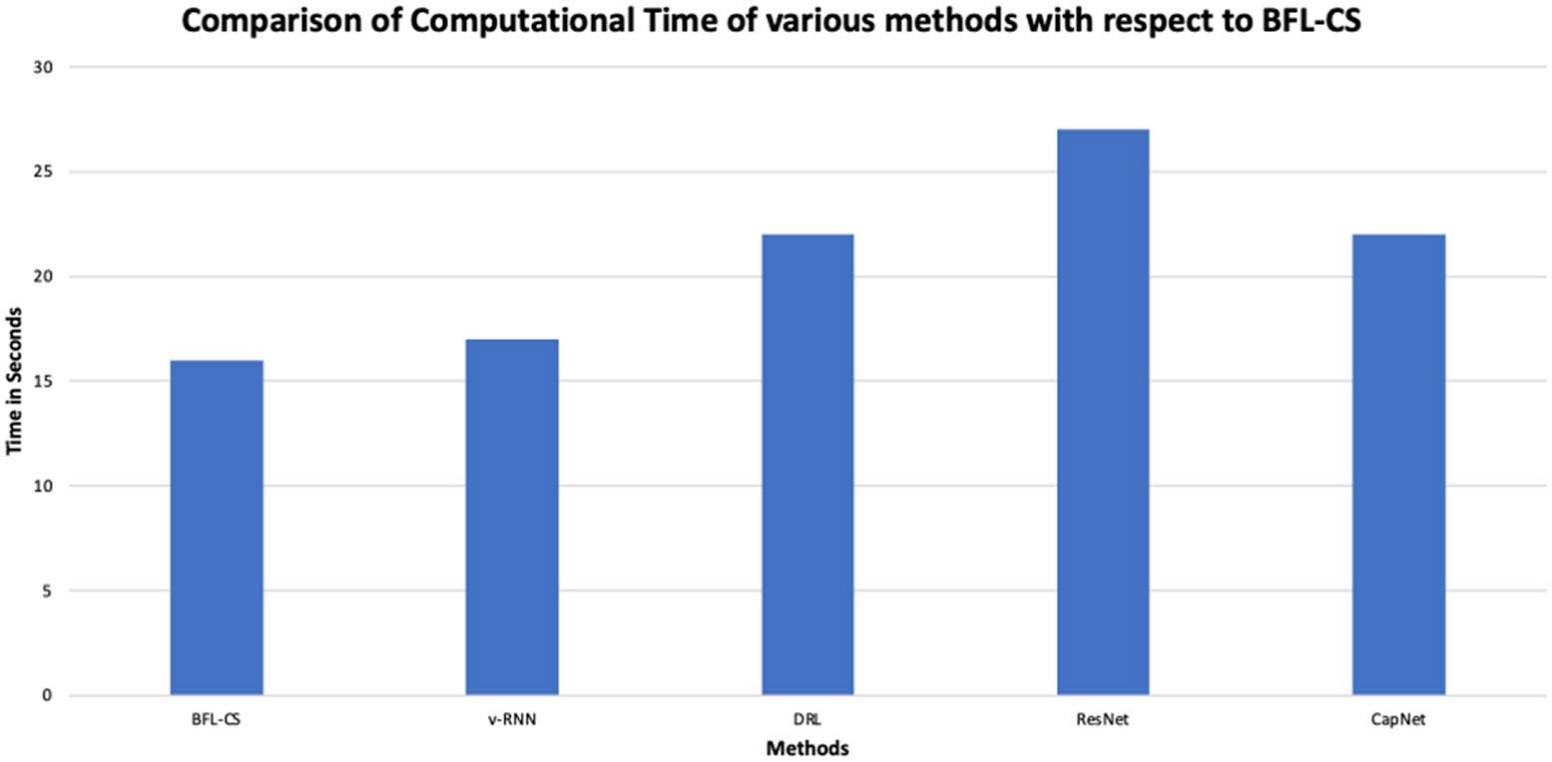
Comparison of run time.

The proposed methodology achieved high specificity of 97.55% while the existing methods obtained low specificity of 96.37, 95.61, 94.53, and 94.16%, respectively (Figures 7, 8).

In above plot shows the area under curve of the proposed methodology. The proposed solution method showed a higher ROC of 0.9812 while the existing models such as vRNN, DRL, ResNet, and CapNet obtained a low AUC-ROC of 0.9691, 0.9592, 0.9494 and 0.9576, respectively.

We have tabulated the comparison of various statistical parameters of the proposed solution and the existing models such as vRNN, DRL, ResNet and CapNet. The details of our analysis are given (Table 1).

**Table 1:** Tabulation of statistical performance measure of various laid down processes against the proposed methodology.

Methods	Accuracy (%)	Precision (%)	Recall (%)	F1-score (%)	Specificity (%)	AUC-ROC	Computational Time (seconds)
BFL-CS	98.92	97.91	97.82	97.86	97.85	0.9812	16
v-RNN	93.21	95.12	96.58	96.42	96.37	0.9721	19
DRL	96.43	96.17	96.13	96.07	95.61	0.9632	22
ResNet	95.38	95.34	95.41	95.34	94.53	0.9574	27
CapNet	97.2	95.12	94.79	94.73	94.16	0.9526	33

BFL-CS achieved the highest accuracy (98.92%) and AUC-ROC (0.9812), indicating that it correctly classified the most data points and has the best ability to distinguish between positive and negative classes. However, it also has the second highest computational time (16 s).

v-RNN, DRL, and ResNet all have similar performance in terms of accuracy (around 95–96%) and computational time (around 20 s). They also have good precision, recall, and F1-score, which means they are good at identifying both positive and negative cases correctly. CapNet has a slightly lower accuracy (97.2%) and AUC-ROC (0.9526) compared to the other methods, but it has the highest computational time (33 s). This suggests that CapNet may be less efficient than the other methods, even though it has a good overall performance. In addition to comparison of BFL-CS with respect to other Deep Learning models, we also compared the accuracy of other implemented solutions (Table 2).

**Table 2:** Comparison of technique, dataset & accuracy of previous work done on the subject.

#	Paper title & Ref No.	Techniques used	Dataset	Accuracy
1	Shetty et al. (3)	SBERT, Universal Sentence Encoders –DAN, Universal Sentence Encoders – Transformers	Data from Kaggle, Youtube, Twitter	97.12%
2	Fati et al. (8)	Continuous Bag of Words based Conv1DLSTM	Data from Kaggle	97.34%
3	Bruwaene et al. (9)	Multi-technique annotation and a ensemble of SVM, CNN & XGBoost	VISR Dataset	–
4	Bozyigit et al. (10)	Artificial Neural Networks	Twitter – Hindi/Marathi	91%
5	Samee et al. (11)	FedBERT	Twitter	92.15%
6	Proposed model	BFL-CS [Blockchain, Federate Learning, Deep Learning (LSTM & DBN in-tandem)].	Cyberbullying dataset	98.2%

The table suggests that models using sentence encoders (SBERT, DAN) perform well on publicly available data (Kaggle, Youtube, Twitter) and achieve high accuracy (above 97%). The model using a multi-technique approach (SVM, CNN, XGBoost) shows competitive performance on a specific dataset (VISR) (37, 38). The BFL-CS model, which combines blockchain, federated learning, and deep learning (LSTM & DBN), achieves the highest accuracy but the data source is not specified.

## 5 Conclusion

The study done on the paper is a novel approach named Blockchain & Federated Learning based Cybersecurity Solution (BFL-CS) Algorithm for detection and prevention of Cyberbullying in social media. In this study, LSTM-DBN in-tandem is utilized along with block chain based federated learning. We see from our design that a major roadblock of the proposed methodology is the usage of multiple technologies in the model, therefore making it very complex for implementation, particularly in implementation of Federated Learning where two complex deep learning methods are already running, while FL is being carried out across the blocks of a real time updated ledger. This level of interconnectedness with various cutting edge technologies will required significant computational resources and strong network data transfer capabilities, however, we have tried to solve this problem by keeping only one epoch of Block-chain updation post training of data, when we increase the frequency of block updations, this approach may prove to very computationally expensive, as each updation will need a hashing process and consensus building. In the future, we should explore in making the blockchain and vanilla federated learning processes more effective. At this point of time, we have high efficacy with respect to the Deep Learning engine, however, this only contributes to only a fraction of what this approach is all about. However, handling the federated learning layer is very crucial when the size of data increases. While there has been attempts in the past at making this process more efficient, however, all of these have created compromises in the security part of it. Therefore, the future scope of work will play out in this direction. In the future scope of work, we try in developing an in-line module in one of the social networks to do a real time reporting and correction of cyberbullying online.

## Data availability statement

The original contributions presented in the study are included in the article/supplementary material, further inquiries can be directed to the corresponding author.

## Author contributions

AA: Conceptualization, Formal analysis, Funding acquisition, Methodology, Project administration, Writing – original draft. AM: Conceptualization, Formal analysis, Data curation, Validation, Visualization, Writing – review & editing.
